# Tocolysis in the management of preterm prelabor rupture of membranes at 22–33 weeks of gestation: study protocol for a multicenter, double-blind, randomized controlled trial comparing nifedipine with placebo (TOCOPROM)

**DOI:** 10.1186/s12884-021-04047-2

**Published:** 2021-09-08

**Authors:** Elsa Lorthe, Gilles Kayem, on behalf of the TOCOPROM Study Group and the GROG (Groupe de Recherche en Obstétrique et Gynécologie), Gilles Kayem, Elsa Lorthe, Pierre-Yves Ancel, Hendy Abdoul, Nelly Briand, Blandine Lehmann, Clémence Cabanne, Stéphane Marret, Laurence Foix l’Hélias, François Goffinet, Thomas Schmitz, Caroline Charlier, Fanny Autret, Elie Azria, Jadot Balitalike, Kareen Billiemaz, Caroline Bohec, Pascal Bolot, Marie Bornes, Hanane Bouchghoul, Malek Bourennane, Florence Bretelle, Lionel Carbillon, Christine Castel, Céline Chauleur, Romain Corroenne, Karen Coste, Valérie Datin-Dorrière, Raoul Desbriere, Luc Desfrere, Michel Dreyfus, Marc Dommergues, Xavier Durrmeyer, Géraldine Favrais, Cyril Flamant, Denis Gallot, Julie Gries, Bassam Haddad, Laure Julé, Cécile Laffaille, Jacques Lepercq, Emmanuelle Letamendia, Fanny de Marcillac, Caroline Miler, Olivier Morel, Karine Norbert, Franck Perrotin, Christophe Poncelet, Laurent Renesme, Claire Roumegoux, Patrick Rozenberg, Mireille Ruiz, Loïc Sentilhes, Jeanne Sibiude, Damien Subtil, Nadia Tillouche, Héloïse Torchin, Barthélémy Tosello, Eric Verspyck, Alexandre Vivanti, Norbert Winer

**Affiliations:** 1 Université de Paris, Epidemiology and Statistics Research Center/CRESS, INSERM, INRA, F-75004 Paris, France; 2 Unit of Population Epidemiology, Department of Primary Care Medicine, Geneva University Hospitals, 1205 Geneva, Switzerland; 3 Department of Gynecology and Obstetrics, Trousseau Hospital, APHP, FHU Prema, Sorbonne University, Paris, France

**Keywords:** Tocolysis, Nifedipine, Randomized controlled trial, Pregnancy, Preterm birth, Preterm prelabor rupture of membranes, Neonatal outcome

## Abstract

**Background:**

Preterm prelabor rupture of membranes (PPROM) before 34 weeks of gestation complicates 1% of pregnancies and accounts for one-third of preterm births. International guidelines recommend expectant management, along with antenatal steroids before 34 weeks and antibiotics. Up-to-date evidence about the risks and benefits of administering tocolysis after PPROM, however, is lacking. In theory, reducing uterine contractility could delay delivery and reduce the risks of prematurity and its adverse short- and long-term consequences, but it might also prolong fetal exposure to inflammation, infection, and acute obstetric complications, potentially associated with neonatal death or long-term sequelae. The primary objective of this study is to assess whether short-term (48 h) tocolysis reduces perinatal mortality/morbidity in PPROM at 22 to 33 completed weeks of gestation.

**Methods:**

A randomized, double-blind, placebo-controlled, superiority trial will be performed in 29 French maternity units. Women with PPROM between 22^0/7^ and 33^6/7^ weeks of gestation, a singleton pregnancy, and no condition contraindicating expectant management will be randomized to receive a 48-hour oral treatment by either nifedipine or placebo (1:1 ratio). The primary outcome will be the occurrence of perinatal mortality/morbidity, a composite outcome including fetal death, neonatal death, or severe neonatal morbidity before discharge. If we assume an alpha-risk of 0.05 and beta-risk of 0.20 (i.e., a statistical power of 80%), 702 women (351 per arm) are required to show a reduction of the primary endpoint from 35% (placebo group) to 25% (nifedipine group). We plan to increase the required number of subjects by 20%, to replace any patients who leave the study early. The total number of subjects required is thus 850. Data will be analyzed by the intention-to-treat principle.

**Discussion:**

This trial will inform practices and policies worldwide. Optimized prenatal management to improve the prognosis of infants born preterm could benefit about 50,000 women in the European Union and 40,000 in the United States each year.

**Trial registration:**

ClinicalTrials.gov identifier: NCT03976063 (registration date June 5, 2019).

**Supplementary Information:**

The online version contains supplementary material available at 10.1186/s12884-021-04047-2.

## Background

### Rationale

#### Epidemiology of PPROM and neonatal consequences

Preterm prelabor rupture of membranes (PPROM) is defined as spontaneous rupture of the fetal membranes occurring before the onset of labor and before 37 weeks of gestation [1, 2]. PPROM is a complex and multifactorial pathology, resulting from the progressive weakening of the membranes under the effect of chemical, mechanical, and/or infectious factors [3]. This degeneration begins several weeks before manifesting clinically by the flow of amniotic fluid [4, 5]. Primary and secondary prevention of preterm birth are very challenging: although some risk factors have been identified, most women have none, and there are no validated predictive models and no effective preventive interventions [6, 7].

PPROM complicates 2–3% of pregnancies [1, 7]. Despite the rupture of membranes, pregnancy can be prolonged by a latency period (defined as the time elapsed from PPROM to delivery) ranging from a few hours to several weeks. About 50 to 60% of women will give birth within the first week after PPROM [8–10], which is thus responsible for a large share of preterm births (25 to 30%) [1, 11] and is a leading cause of neonatal mortality and morbidity and maternal infectious morbidity [7, 12]. Although the infant’s prognosis depends mainly on gestational age at birth [13–15], fetal exposure to inflammation, infection, and acute obstetric complications (placental abruption, umbilical cord compression or prolapse) can increase short- and long-term mortality/morbidity [16–18]. Consequently, in cases of PPROM, medical teams must weigh the benefits of prolonging pregnancy to reduce prematurity-related adverse consequences against those of inducing delivery to shorten exposure to intrauterine inflammation, infection, and acute obstetric complications.

#### Initial antenatal management

Antenatal management of women follows tertiary prevention principles and aims to reduce maternal, fetal, and neonatal adverse consequences [19]. With PPROM before 34 weeks, current evidence-based care includes expectant management in the absence of labor, overt infection, or fetal distress [20–23] to increase gestational age at birth — the main determinant of the preterm child’s prognosis [13–15]. Routine administration of antibiotics is recommended to prolong pregnancy and reduce neonatal and maternal morbidity [24–27]. The administration of a single course of antenatal steroids is also part of routine care to reduce neonatal mortality, respiratory distress, necrotizing enterocolitis, and intraventricular hemorrhage [28]. Transfer to a tertiary care center should also be offered if necessary [23].

#### A controversial treatment: tocolysis

Tocolysis aims to inhibit uterine contractions and thereby prolong pregnancy at least long enough for a complete course of antenatal steroids and to reduce the consequences of prematurity. However, it can also increase fetal exposure to infection and acute complications.

Only a few randomized controlled trials have addressed the benefits and harms of tocolysis in women with PPROM (Table 1) [29–37]. Overall, they did not demonstrate improvement in neonatal outcomes, and the results regarding the prolongation of gestation remain controversial (Table 1). Most of these trials took place in the 1980s, when steroids and antibiotics were not part of routine care, and performance and reporting biases were not uncommon [12, 38]. Because they were mostly powered to show a difference in latency duration and thus had small sample sizes (6 to 81 patients), their external validity and reliability are limited.

**Table 1:** Characteristics of randomized trials comparing initial tocolysis vs. no tocolysis or placebo in women with PPROM

Author, year (reference)	Methods, n	Inclusion criteria	Intervention	Antibiotics / Steroids	Primary outcome	Main result
Christensen, 1980 [29]	RCT, *n* = 30	Singletons, 28–36 wks, with contractions	Ritodrine vs placebo, until 35 wks	Only for urogenital colonization / not specified	Not pre-specified	Significant reduction of deliveries within 24 h, no difference at 48 h
Levy, 1985 [30]	RCT, *n* = 42	Singletons, 25–34 wks, no contractions	Ritodrine vs placebo, until labor	Only if cesarean section / no	Latency period	Significant prolonged mean latency period for treated women
Dunlop, 1986 [31]	RCT, *n* = 48	Singletons, 26–34 wks, no uterine contractions	A: no ritodrine, no cephalexin B: ritodrine, cephalexin C: ritodrine, no cephalexin D: no ritodrine, cephalexin	Only for groups B and D / systematic	Type of labor, mode of delivery, neonatal and maternal outcomes including admission to birth interval	No advantage to the newborn
Garite, 1987 [32]	RCT, *n* = 79	Singletons, 25–30 wks, with or without contractions	Ritodrine vs placebo, until 31 wks	Only if cesarean section / no	Time interval from PPROM to birth	No difference
Weiner, 1988 [33]	RT, *n* = 75 (+ 34 excluded from analyses)	Singletons, up to 34 wks, with contractions	Ritodrine, terbutaline or magnesium sulfate vs bedrest, no clear duration	Only for urogenital colonization / no	Not clearly stated	No difference
Matsuda, 1993 [34]	RT, *n* = 81	Singletons, 23–34 wks, no contractions	Ritodrine vs bedrest, no clear duration	Only for treated women / not specified	Prolongation of pregnancy	Prolongation for more than 72 h was greater for treated women
How, 1998 [35]	RCT, *n* = 145	Singletons and twins, 24–34 wks, not in labor	Magnesium sulfate (treatment initiated only if contractions occurred) vs no tocolysis	Systematic / systematic (weekly)	Not clearly stated	No difference in latency duration, no difference in neonatal outcomes
Ehsanipoor, 2011 [36]	RCT, *n* = 47	Singletons, 24–31 w, no contractions	Indomethacin vs placebo, for 48 h	Systematic / systematic	Delivery within 48 h	No difference
Nijman, 2016 [37]	RCT, *n* = 50	Singletons and twins, 24–33 wks, no contractions	Nifedipine vs placebo, until the onset of labor (up to 18 days or 34 wks)	According to local guidelines / Systematic	Composite of poor neonatal outcome	No difference

*h* hours, *RCT* randomized controlled trial, *RT* randomized trial, *wks* weeks

A systematic review including 8 trials and 408 women compared the potential benefits and harms of any tocolytic therapy with no tocolytic, placebo, or another tocolytic [12]. It found that tocolysis was associated with a significant prolongation of gestation (mean difference 73 h; 95% confidence interval [CI] 20–126; three trials of 198 women) and fewer births within 48 h. Neonatal morbidity (5-min Apgar < 7 and greater need for ventilation) increased as did maternal chorioamnionitis, with no benefits to the infant. The authors concluded that further evaluation of tocolysis is required in women with PPROM treated according to current standards of care.

Two recent studies investigated the association of tocolysis with neonatal outcomes and latency duration based on observational population-based cohort data; they used various approaches to minimize indication bias [38, 39]. Both suggested that tocolysis in PPROM was not associated with improved obstetric, neonatal, or two-year outcomes of preterm infants. Nonetheless, as most clinical guidelines acknowledge, current data are insufficient to support or refute initial tocolysis in women with PPROM [21, 22]. Clinical practices therefore vary widely [40–43].

#### The choice of nifedipine

Several tocolytics have been tested to stop contractions in preterm labor (PTL). In this setting, betamimetics and calcium channel blockers (CCBs) have demonstrated a benefit over placebo in prolonging pregnancy [44, 45], while oxytocin receptor antagonists have not been shown to be superior to placebo, betamimetics, or CCBs (principally nifedipine) for either pregnancy prolongation or neonatal outcomes [46].

For the TOCOPROM trial, we have chosen to use nifedipine, a CCB with a nonspecific relaxant effect on smooth muscles. Although it has rarely been studied for PPROM, nifedipine is commonly recommended and used in France and worldwide for first-line tocolysis to treat spontaneous PTL [47, 48]. Its additional advantages include its favorable safety profile, with severe maternal adverse effects reported only rarely (contrary to betamimetics), and no described fetal or infant complications, as well as oral administration and a reasonable cost (compared with atosiban) [45]. Finally, given its lack of marketing authorization for the treatment of PTL or PPROM, the Pharmacovigilance Technical Committee of the French National Agency of Drug Safety (ANSM) delivered a temporary use recommendation in 2015.

#### Trial aims

The primary objective is to prospectively assess if short-term (48 h) tocolysis reduces perinatal mortality/morbidity in women with PPROM at 22 to 33 completed weeks of gestation by performing a multicenter, randomized, double-blind, placebo-controlled trial.

Its secondary objectives are to assess the impact of tocolysis on prolongation of gestation, maternal morbidity, neonatal morbidity, and outcomes at 2 years of corrected age in cases of PPROM at 22 to 33 weeks.

## Methods and design

### Study design

The TOCOPROM study will be a double-blind, randomized, controlled, superiority, phase III trial comparing two parallel groups receiving a 48-h oral treatment by either nifedipine or placebo (1:1 ratio). The trial protocol (current version 5.0, April 2021) follows the framework of the clinical research and innovation department (Délégation à la Recherche Clinique et à l’Innovation, DRCI, Assistance Publique – Hôpitaux de Paris), which adheres to the Standard Protocol Items: Recommendations for Interventional Trials (SPIRIT) 2013 Statement for protocols of clinical trials [49].

### Setting

This nationwide multicenter trial involves 29 departments of obstetrics and gynecology in France: 24 level III and 5 level II maternity units (Supplementary Table 1).

### Inclusion criteria

Pregnant women are eligible for the trial if they meet all of the following criteria:

- PPROM, diagnosed by obstetric teams, between 22^0/7^ and 33^6/7^ weeks of gestation, dated according to the first-trimester ultrasonography.
- Singleton pregnancy.
- Fetus alive at randomization.
- Age ≥ 18 years.
- Speaks French.
- Affiliated with the French national health insurance or an equivalent system.
- Provided written informed consent.

### Exclusion criteria

Pregnant women are not eligible for the trial if they meet any of the following criteria:

- PPROM diagnosis ≥24 h after amniotic fluid leak.
- Ongoing tocolytic treatment at the time of PPROM.
- Tocolytic treatment with nifedipine between PPROM diagnosis and randomization (*before July 16, 2021, this criterion was “Any tocolytic treatment between PPROM diagnosis and randomization”. This modification was introduced after randomizing 211 patients and was intended to improve the potential for inclusion by making patients receiving a brief tocolysis during* in utero *transfer eligible.)*
- Fetal condition contraindicating expectant management (including intrauterine infection, placental abruption, hemorrhagic placenta previa, intrauterine fetal demise, non-reassuring fetal heart rate).
- Cervical dilation ≥5 cm.
- Iatrogenic rupture of membranes caused by amniocentesis or trophoblast biopsy.
- Major fetal anomaly.
- Maternal allergy or contraindication to nifedipine or placebo drug components (myocardial infarction, unstable angina pectoris, hepatic insufficiency, cardiovascular shock, beta blockers, and cardiopathy).
- Co-administration of diltiazem, rifampicin, transdermal nitrates, or any antihypertensive medication.
- Hypotension (systolic blood pressure < 90 mmHg).
- Participation in another interventional research study (Category 1).

### Study intervention

The intervention will be the oral administration of either nifedipine 20 mg or placebo of nifedipine 20 mg (according to the randomization group) for 48 h according to the following administration protocol:

- Loading dose: 1 tablet at Time 0 (T0) and T0.5 (i.e., 30 min after the first intake),
- Maintenance dose: 1 tablet at T3, T11, T19, T27, T35, and T43.

The total treatment duration will be 48 h, with nifedipine total doses of 100 mg on day 1 and 60 mg on day 2.

### Study procedures

#### Recruitment

Each woman presenting at a participating center with possible PPROM will be examined by a midwife or an obstetrician to confirm or rule out this diagnosis. PPROM diagnosis, according to national clinical guidelines, is usually based on 2 positive criteria from maternal history, sterile speculum examination to confirm fluid leakage from the cervical canal, and performance of a diagnostic test [21]. Women transferred from another center will be eligible if PPROM occurred within the previous 24 h and if they did not receive nifedipine after the PPROM diagnosis (women who received other tocolytics during in utero transfer, such as atosiban, which has a very short half-life, are eligible from July 16, 2021 onwards).

Eligible women who meet inclusion criteria will be identified by a local investigator (obstetrician or midwife) and informed about the trial (orally and with a written patient information form) (Table 2). Those who choose to participate, after a reflection period if necessary, will provide written informed consent. A second informed consent form, signed by the mother and father (at inclusion, delivery, or follow-up assessment), is required for the follow-up of children at 2 years of corrected age.

**Table 2:**
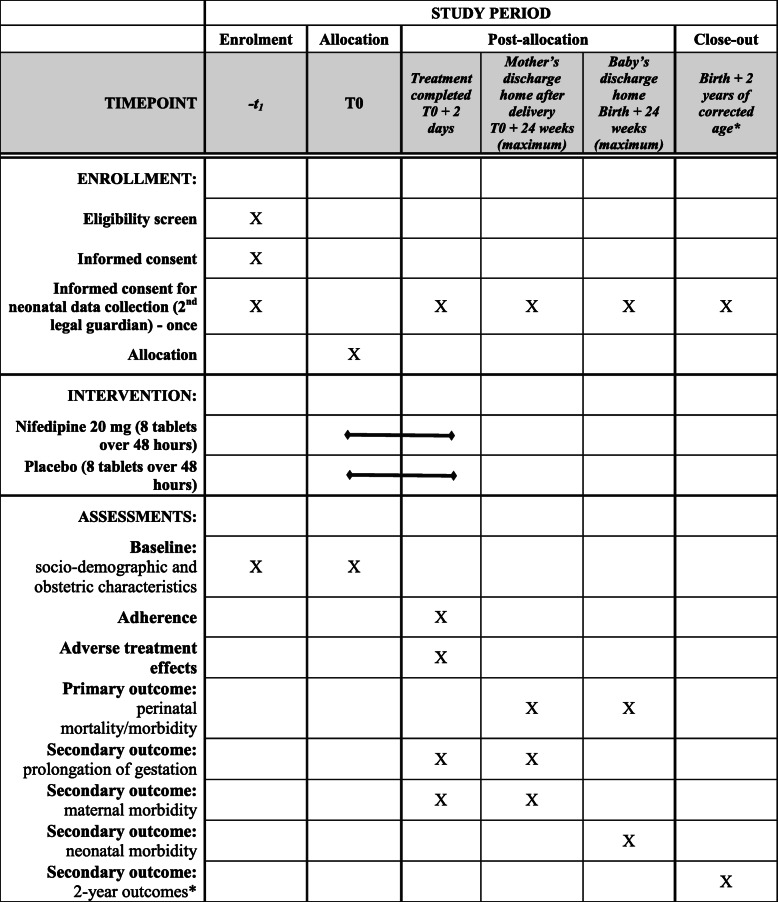
Schedule of enrollment, intervention, and assessment in the TOCOPROM trial

* A follow-up evaluation at the age of 5 years is under consideration, if additional funding can be obtained

#### Allocation of treatment

After the local investigator (an obstetrician or a midwife) has obtained maternal consent and immediately after inclusion in the trial, he or she will use a centralized procedure provided by a web-based computerized program with secure access (CleanWEB™ software) to randomize the patient. The randomization procedure will use minimization, a dynamic method that allocates subjects to the treatment group that best maintains balance in stratifying factors [49, 50]. Thus, the randomization list is not produced before the trial starts, but during participant recruitment. Minimization will be based on three prognostic factors: uterine contractions felt by the patient at the time of inclusion (presence or absence), recruiting center (1 to 29), and gestational age at PPROM (22/23 weeks, 24/26 weeks, 27/30 weeks, 31/33 weeks). The first 50 patients will be assigned through simple randomization. For the next step, the marginal totals in each treatment arm for the prognostic factors of the 51st patient will be calculated; the objective is to balance these marginal totals. The 51st patient will be assigned to the arm that improves the balance according to the preselected set of factors between the 2 trial arms. The same process will be applied for each new patient. 10% of randomness will be incorporated into the minimization algorithm, to make the prediction unlikely.

Each woman will receive a unique identification number after inclusion and a treatment box number after randomization.

#### Treatment administration

The allocated treatment box, containing 8 tablets of nifedipine or placebo, will be immediately given to the patient by the local investigator or by his/her delegate, along with a patient compliance form specifying the theoretical timing of each drug intake. The patient will start the protocol as soon as possible with the first intake. Within the first hour after starting nifedipine or placebo, blood pressure, heart rate, and clinical symptoms (such as dyspnea or thoracic pain) will be measured according to each maternity unit’s usual practices. Treatment will continue as long as blood pressure remains within normal limits, with blood pressure and heart rate thereafter measured 2 to 3 times per day. Clinical surveillance and management during the protocol will take place according to local practices, without any additional procedures or data collection. The treatment can be interrupted by the patient and/or the investigator for any reason deemed necessary (including the need for rescue tocolysis).

After each intake, the patient will report its actual date and time on the compliance form, as well as any side effects and comments. After the protocol is completed, the local investigator will collect the treatment box and blister pack and send them to the local pharmacy.

#### Blinding and unblinding procedures

Patients, healthcare providers, pharmacists, and investigators will be blinded throughout the trial. The experimental medication comprises tablets of NIFEDIPINE MYLAN® LP 20 mg - nifedipine. Placebo pink tablets of nifedipine 20 mg will be specifically formulated, manufactured, and packaged (primary packaging: neutral PVC/PVDC/Aluminium blisters) under the responsibility of the Clinical Trials Department (Département Essais Cliniques, DEC) of the General Agency for Health Equipment and Products (Agence Générale des Equipements et Produits de Santé, AGEPS). Nifedipine and placebo will be packed by the Clinical Trials Department in strictly identical numbered sealed boxes, containing one blister pack of 8 tablets of nifedipine 20 mg or placebo.

Unblinding will be requested for any reason considered essential by the investigating doctor by contacting the relevant departments, i.e., the DRCI or the Fernand Widal Hospital poison center (in emergency situations or outside of usual working days and hours).

#### Maternal management and follow-up

Women’s management will otherwise be identical to that in routine practice for PPROM. Usual practices include hospitalization for at least 48 h after the PPROM diagnosis and then hospitalization or home hospitalization until delivery. Except for the experimental intervention, obstetric management (including medical, laboratory, and paraclinical examinations) and treatments (antibiotics, antenatal steroids, and magnesium sulfate) will be based on local protocols and medical teams’ decisions.

Women will be followed up from randomization to discharge home after delivery.

#### Neonatal management and follow-up

Neonatal management will be exactly the same as it would be in routine practice. Infants will be followed from birth to discharge home or until a maximum of 24 weeks of age, whichever comes first.

#### Child follow-up

We will perform a follow-up at 2 years of corrected age, with a self-administered questionnaire sent to the parents, by email and/or postal mail, by using the contact details they provided at inclusion. Besides severe morbidity considered as a secondary endpoint, we will evaluate the children’s growth, health, neurodevelopment, and living conditions (family situation and socioeconomic conditions). Another follow-up evaluation at the age of 5 years is under consideration, if additional funding can be obtained.

#### Duration of the trial

The total duration of the trial will be 71 months, including 42 months of inclusions and the follow-up at 2 years of corrected age (i.e., for a maximum of 29 months for infants born extremely preterm).

### Outcome measures

#### Primary outcome measure

The primary endpoint is a composite outcome of perinatal mortality/morbidity until hospital discharge (or up to a maximum of 24 wks after birth if the infant is still hospitalized). It includes fetal death (in utero fetal death occurring from randomization to birth), neonatal death (death from birth to discharge, in the delivery room or neonatal intensive care unit [NICU]), and/or severe neonatal morbidity [51] defined as any one or more of the following:

- Mechanical ventilation ≥48 h, defined as high frequency or conventional mechanical ventilation for at least 48 consecutive hours during hospitalization [52];
- Severe bronchopulmonary dysplasia (BPD), defined as requiring oxygen for at least 28 days plus the need for 30% or more oxygen and/or mechanical ventilator support or continuous positive airway pressure at 56 days postnatal age or 36 weeks’ postmenstrual age or discharge, whichever comes first [53];
- Severe intraventricular hemorrhage (IVH), defined as IVH associated with ventricular dilatation (grade III IVH) and intraparenchymal hemorrhage (i.e., large unilateral parenchymal hyperdensity or large unilateral porencephalic cyst, grade IV IVH); diagnosed by ultrasound [54];
- Cystic periventricular leukomalacia, defined as periventricular white matter echolucencies on ultrasonography [55];
- Early-onset sepsis, diagnosed from positive bacteriology findings in blood or cerebrospinal fluid (confirmed infection) that began in the first 3 days of life [56];
- Necrotizing enterocolitis, stages II and III according to Bell’s staging [57];
- Retinopathy of prematurity, stage 3 or greater according to the international classification [58] and/or laser treatment.

#### Secondary outcome measures

Prolongation of gestation will be evaluated by various different criteria:

- Latency duration, defined as the interval from PPROM to delivery.
- Pregnancy prolongation beyond 48 h after randomization,
- Pregnancy prolongation beyond 1 week after randomization,
- Gestational age at delivery,
- Delivery after 37 weeks of gestation.

Maternal morbidity will be assessed by clinical diagnosis of intrauterine infection, defined as fever (maternal temperature ≥ 38 °C), with no alternative cause identified, associated with at least two of the following criteria: persistent fetal tachycardia > 160 bpm, uterine pain or painful uterine contractions or spontaneous labor, purulent amniotic fluid [21]; and endometritis, based on the association of fever (temperature ≥ 38.0 °C) with uterine tenderness, purulent or foul-smelling lochia, and in the absence of any other cause, during the first 10 days after delivery [59].

For neonatal morbidity, we will isolate each criterion included in the composite primary outcome and assess its association with the intervention. We will also study:

- Severe fetal acidemia, defined as cord umbilical artery pH less than 7.00 or base deficit greater than 16 mEq/L (16 mmol/L), or both [60];
- Respiratory distress syndrome, defined from clinical diagnosis as the presence of clinical signs of respiratory distress (tachypnea, retractions, flaring, grunting, or cyanosis), with a requirement for supplemental oxygen with a fraction of inspired oxygen more than 0.21 and a chest radiograph showing hypoaeration and reticulogranular infiltrates;
- Mild or moderate BPD, defined as requiring oxygen for at least 28 days plus the need for 21% (mild) - 30% (moderate) oxygen at 56 days postnatal age or 36 weeks’ postmenstrual age or discharge, whichever comes first [53];
- Grades I-II IVH, defined according to Papile’s classification: subependymal hemorrhage (grade I IVH) or IVH without ventricular dilatation (grade II IVH), diagnosed by ultrasound [54];
- Late-onset sepsis, diagnosed from positive bacteriology findings in blood or cerebrospinal fluid (confirmed infection) that began after 72 h of life [61].

At 2 years of corrected age, we will evaluate the following criteria [51]:

- Vital status (ascertainment of whether the child died between discharge and follow-up at 2 years or is still alive);
- Cerebral palsy, defined according to the diagnostic criteria of the Surveillance of Cerebral Palsy in Europe (SCPE) network [62], with severity graded with the five-level Gross Motor Function Classification System (GMFCS) [63];
- Hearing impairment, defined as deafness or functional hearing loss requiring correction and classified as severe (bilateral) or moderate (unilateral) [64];
- Visual impairment, defined as blindness or the ability to see light only, and classified as severe (bilateral) or moderate (unilateral) [64]. Squinting or the need for glasses will also be recorded;
- Neurodevelopment, assessed with the second version of the 24-month Ages and Stages Questionnaire (ASQ), which is validated in France and covers five developmental domains: communication abilities, gross motor skills, fine motor skills, problem-solving abilities, and personal-social skills [64, 65].

### Data collection, management, and monitoring

Maternal adverse events will be recorded prospectively from the first nifedipine or placebo intake to the end of follow-up. Maternal and obstetric data will be available in medical files and will be recorded on the electronic case report form (e-CRF) after the end of the follow-up period. Although this situation is unlikely, if a patient delivers outside the recruiting maternity unit, a duplicate of the medical record will be requested to collect all necessary data.

Neonatal deaths will be reported prospectively. All neonatal data will be recorded on the e-CRF based on medical files, after the infant’s discharge. In NICUs, hospital reports are very precise and state clearly all pathologies and complications diagnosed during the stay. Diagnoses generally use international definitions and are therefore standardized. If any neonate is transferred to a NICU outside of the recruiting center, the investigators will request the hospital report and/or a duplicate of the medical file to collect all necessary data.

This study, including quality controls, will be conducted according to the standard operating procedures of the sponsor, Assistance Publique – Hôpitaux de Paris (AP-HP), the Declaration of Helsinki, and Good Clinical Practices. All data will be recorded by local investigators, research midwives, or trained clinical research technicians on a secure eCRF. Local investigators will be responsible for the accuracy, quality, and relevance of all the data entered. Specific codes will be entered for data missing in the medical records, to distinguish them from data entry errors. Cross-checks for completeness and consistency checks will take place periodically.

Data management will be handled centrally by the Clinical Research Unit (Paris Descartes Necker Cochin) with the study’s Scientific Directors (EL, PYA). A data management plan will be written and followed throughout the entire data management and analysis process. Clinical research assistants will organize periodic contacts and monitoring visits to each recruiting center. These will include a telephone contact within 2 weeks of the first inclusion, an onsite visit within 3 months of the first inclusion, and then a visit every 10 inclusions or once a year, whichever comes first. Compliance with the research protocol and procedures, consent forms, and predefined relevant data (eligibility criteria, adherence, primary and secondary outcome measures, and severe adverse events with immediate notification) will be monitored onsite, while severe adverse events without immediate notification and 2-year outcomes will be monitored remotely. All study documents will be archived by the investigators and the sponsor for 15 years after the end of the trial.

### Confidentiality and data handling

Data will be handled according to the French (amended “Informatique et Libertés” law governing data protection) and European (General Data Protection Regulation, GDPR) regulations. The eCRFs will be hosted by a service provider in a secure electronic system via a web navigator and protected by an individual password for each investigator and clinical research technician.

The full identity of the research participants and their contact details will be recorded for purposes of the 2-year follow-up, with the authorization of the French Data Protection Agency (CNIL, Reference 919221, August 2, 2019). Identity and contact details will be kept separately from clinical data. To ensure confidentiality, participant’s identifying information will be replaced by an identification code specific to the study indicating the order of enrolment.

The steering committee will have access to the full anonymized trial dataset. The trial database file will be stored for 15 years. The sponsor is the owner of the data.

### Statistical issues

#### Sample size

Within the prospective, national, population-based EPIPAGE-2 cohort study of preterm births, we selected a sample of 888 women according to the TOCOPROM eligibility criteria and estimated the frequency of infants diagnosed with any criterion of the composite primary outcome to be 35.5%. This is an average estimate taking into account the expected variation of gestational age at birth in our study population from 22 to 37+ weeks. This estimate is consistent with findings in recent studies [15, 52, 66].

We hypothesized that the beneficial effect of tocolysis would be mediated by both higher gestational age at birth and the administration of a complete course of antenatal corticosteroids. The EPIPAGE-2 study showed that among women with PPROM, each one-day increase in gestational age at birth is significantly associated with a reduced relative risk (RR) of fetal or neonatal death or severe morbidity (RR 0.95, 95% CI 0.91–0.99) [10]. With pregnancy prolonged by 48 h by effective tocolysis, we expect to reduce the rate of fetal or neonatal death or severe morbidity by 10%. Each additional day after the initial 48-h period will also contribute to reducing adverse outcomes. Moreover, this initial prolongation will allow a complete course of antenatal corticosteroids, with additional beneficial effects, as shown by Roberts et al. [28, 67]. In meta-analyses of women with PPROM, antenatal steroids have been associated with reduced perinatal (RR 0.59, 95%CI 0.39–0.90) and neonatal mortality (RR 0.61, 0.46-0.83), respiratory distress syndrome (RR 0.70, 0.55-0.90), chronic lung disease (RR 0.50, 0.33–0.76), necrotizing enterocolitis (RR 0.39, 0.18–0.86), and IVH (RR 0.47, 0.28-0.79). Retinopathy of prematurity, early-onset sepsis, and periventricular leukomalacia were not analyzed separately by membrane status in these meta-analyses.

When we assume an alpha-risk of 0.05 and a beta-risk of 0.20 (i.e., statistical power of 80%), 702 women (351 per arm) are required to show a reduction of the primary endpoint from 35% (placebo group) to 25% (nifedipine group). We plan to increase the required number of subjects by 20%, to replace patients who leave the study early. The total number of subjects required is thus 850.

#### Statistical analysis

Data analysis and reporting will follow the CONSORT guidelines for randomized controlled trials [68] and will be conducted according to the following principles. The trial statistician and researchers will be blinded to group status. No intermediate analysis is planned, as there is no added risk for women and neonates from this study compared with routine practices in France. Data will be analyzed by the intention-to-treat principle, i.e., all randomized participants will be analyzed according to their original allocation, regardless of protocol adherence. Baseline demographic and clinical characteristics of women, including adherence to the protocol, and outcomes will be described and compared by allocated treatment. Categorical variables will be summarized by numbers and percentages of patients in each treatment group and compared by Chi-2 or Fisher’s exact tests, as appropriate. Quantitative variables will be presented as means (standard deviations) if their distribution is normal and medians (interquartile ranges) otherwise; they will be compared with Student or Mann-Whitney-Wilcoxon tests, as appropriate. All these statistical tests will be two-sided and the level of statistical significance will be set at 5% (2-sided).

The effects of tocolysis will be expressed as relative risks with their 95% CIs for categorical outcomes and as mean differences with their 95% CIs for quantitative outcomes. Risk ratios and 95% CIs will be calculated for the primary outcome by using Poisson regression with a robust variance estimation, first unadjusted and then adjusted for minimization factors. Center will be fitted as a random effect, and the other minimization factors as fixed effects. The results will also be expressed as absolute risk differences with 95% CIs for binary outcomes. We will assess the Number Needed to Treat (NNT), defined as the number of patients who must be treated to prevent one additional adverse outcome.

Secondary outcome measures will be approached similarly to the primary outcome measure. Latency duration will be evaluated by Kaplan-Meier estimators and compared between the two treatment groups with the Log rank test and Cox proportional hazards regression, adjusted for gestational age at PPROM, if the proportional risks hypothesis is verified.

As a secondary analysis, we will analyze a per-protocol population, namely, women who received the full experimental treatment (eight tablets of nifedipine or placebo).

Characteristics of participants with and without missing data will be compared. If missing data are considered to be missing at random, we will perform multiple imputations with chained equations, with a logistic imputation model for binary covariates and a multinomial imputation model for categorical variables. Otherwise, participants with missing data will be excluded from analyses.

#### Subgroup analyses

Based on the approach previously described, planned subgroup analyses include:

1. Presence or absence of uterine contractions at randomization
2. Gestational age at PPROM (22/23 weeks, 24/26 weeks, 27/30 weeks, and 31/33 weeks)
3. Whether or not any tocolytic treatment was administered between PPROM and randomization.

### Trial steering committee

A trial steering committee, composed of the principal investigator, an epidemiologist, a coordinating midwife, several investigators, and external medical doctors, will be set up to provide overall supervision of the trial. It will meet before the trial starts to approve the final protocol and then whenever it is deemed necessary by the coordinating investigator.

### Safety monitoring

An independent Data Safety Monitoring Board (DSMB) was not deemed necessary for this trial, as nifedipine is routinely used during pregnancy in cases of spontaneous preterm labor or PPROM and has a temporary use recommendation from the National Agency for Drug Security. However, a few conditions (preterm birth, systematic hospitalization, spontaneous labor, labor induction or cesarean section, early- and late-onset sepsis) will be extracted from the database every 6 months and monitored by the Safety Department.

### Ethics

The study protocol and the statistical plan were written before the starting of the trial and approved by the steering committee and the sponsor. Ethics approval was granted by the Committee for the Protection of People participating in biomedical research Sud Méditerranée IV (CPP, Reference 190509, June 11, 2019) and the National Agency for Drug Security (ANSM, Reference MEDAECNAT-2019-06-00048, August 14, 2019). Women will be included and randomized in the TOCOPROM trial only after receiving adequate information and providing written free and informed consent. The second legal guardian’s consent to neonatal and 2-year (and 5-year) data collections will be obtained at maternal inclusion or delivery or at 2 years at the latest. The French Data Protection Agency approved the collection of the full identity and contact details of the research participants for these follow-up purposes (CNIL, Reference DR-2019-171, August 2, 2019).

### Dissemination policy

Results of this trial will be presented at national and international conferences, targeting clinicians and researchers, and published in a peer-reviewed journal, according to the dissemination plan determined by the steering committee. Authorship for manuscripts submitted for publication will follow the criteria defined by the International Committee of Medical Journal Editors.

## Discussion

Available evidence from randomized controlled trials is of insufficient quality to recommend for or against the use of a short-course of tocolysis for women with PPROM. Its effects have never been correctly assessed in women managed according to current standards of care and based on trials adequately powered to show a difference in neonatal outcomes. Nifedipine is a promising candidate drug, inexpensive and easy to administer, that could be further implemented worldwide should this trial demonstrate a beneficial effect.

We hope to inform practices and policies with the results of this large, adequately powered, multicenter, randomized, placebo-controlled trial. Every year, about 50,000 women in the European Union (including 8000 in France) and 40,000 in the United States experience PPROM before 34 weeks. They could benefit from optimized antenatal management to improve the prognosis of infants born preterm.

## Supplementary Information

**Additional file 1 Supplementary Table 1**. List of study sites.

## Data Availability

The datasets generated and/or analyzed during the current study are available from the corresponding author on reasonable request.

## Abbreviations

AGEPS: Agence Générale des Equipements et Produits de Santé (General Agency for Health Equipment and Products)
ANSM: Agence Nationale de Sécurité du Médicament et des produits de santé (National Agency for Drug Security)
AP-HP: Assistance Publique – Hôpitaux de Paris (Paris Public Hospital agency)
ASQ: Ages and Stages Questionnaire
BPD: Bronchopulmonary dysplasia
CCB: Calcium channel blockers
CNIL: Commission Nationale de l’Informatique et des Libertés (French Data Protection Agency)
CONSORT: Consolidated Standards of Reporting Trials
CPP: Committee for the Protection of People participating in biomedical research
DEC: Département Essais Cliniques (Clinical Trials Department)
DRCI: Délégation à la Recherche Clinique et à l’Innovation (Clinical Research and Innovation Department)
DSMB: Data Safety Monitoring Board
EPIPAGE-2: 2nd Etude épidémiologique sur les petits âges gestationnels
GDPR: General Data Protection Regulation
GMFCS: Gross Motor Function Classification System
GROG: Groupe de Recherche en Obstétrique et Gynécologie (Research Group in Obstetrics and Gynecology)
e-CRF: Electronic Case Report Form
IVH: Intraventricular hemorrhage
NICU: Neonatal intensive care unit
NNT: Number needed to treat
PPROM: Preterm prelabor rupture of membranes
PTL: Preterm labor
PVC: Polyvinyl Chloride
PVDC: Polyvinylidene dichloride
RR: Relative risk
SCPE: Surveillance of Cerebral Palsy in Europe
SPIRIT: Standard Protocol Items: Recommendations for Interventional Trials
TOCOPROM: Tocolysis in the management of preterm prelabor rupture of membranes at 22–33 weeks of gestation
