# Supplementary material for: Tocolysis in the management of preterm prelabor rupture of membranes at 22–33 weeks of gestation: study protocol for a multicenter, double-blind, randomized controlled trial comparing nifedipine with placebo (TOCOPROM)
Source: BMC Pregnancy Childbirth. 2021 Sep 8;21:614. doi: 10.1186/s12884-021-04047-2 (PMC8425321; doi:10.1186/s12884-021-04047-2)
Supplement: Supplementary file 1 — Additional file 1 Supplementary Table 1. List of study sites. [file 12884_2021_4047_MOESM1_ESM.docx]

**Supplementary Table 1: List of study sites**

| **Hospital Name** |
| --- |
| CHU d’Angers |
| AP-HP, Jean Verdier, Bondy |
| CHU de Bordeaux |
| CHU de Caen Normandie |
| CHU de Clermont-Ferrand |
| AP-HP, Antoine Béclère, Clamart |
| AP-HP, Louis Mourier, Colombes |
| CHI de Créteil |
| AP-HP, Bicêtre, Le Kremlin-Bicêtre |
| CHU de Lille |
| AP-HM, Hôpital La Conception, Marseille |
| AP-HM, Hôpital Nord, Marseille |
| Hôpital Saint-Joseph, Marseille |
| CHRU de Nancy |
| CHU de Nantes |
| AP-HP, La Pitié Salpétrière, Paris |
| AP-HP, Port-Royal, Paris |
| CH de Saint Denis |
| AP-HP, Tenon, Paris |
| AP-HP, Trousseau, Paris |
| Hôpital Paris Saint-Joseph, Paris |
| CH de Pau |
| CHI de Poissy |
| CH de Pontoise |
| CHU de Rouen |
| CHU de Saint Etienne |
| CHU de Strasbourg |
| CHRU de Tours |
| CH de Valenciennes |

AP-HM: Assistance publique - Hôpitaux de Marseille, AP-HP: Assistance publique - Hôpitaux de Paris, CH: centre hospitalier, CHI: centre hospitalier intercommunal, CHRU: centre hospitalier regional universitaire, CHU: centre hospitalier universitaire
